# Vaccination of Icelandic Children with the 10-Valent Pneumococcal Vaccine Leads to a Significant Herd Effect among Adults in Iceland

**DOI:** 10.1128/JCM.01766-18

**Published:** 2019-03-28

**Authors:** Sigríður J. Quirk, Gunnsteinn Haraldsson, Martha Á. Hjálmarsdóttir, Andries J. van Tonder, Birgir Hrafnkelsson, Stephen D. Bentley, Ásgeir Haraldsson, Helga Erlendsdóttir, Angela B. Brueggemann, Karl G. Kristinsson

**Affiliations:** a University of Iceland, Faculty of Medicine, Reykjavík, Iceland; b Landspitali, University Hospital, Reykjavík, Iceland; c BioMedical Centre of the University of Iceland, Reykjavík, Iceland; d Parasites and Microbes, Wellcome Sanger Institute, Hinxton, United Kingdom; e University of Iceland, Department of Mathematics, Reykjavík, Iceland; f Children’s Hospital Iceland, Reykjavík, Iceland; g Nuffield Department of Medicine, University of Oxford, Oxford, United Kingdom; h Department of Medicine, Imperial College London, London, United Kingdom; University of Iowa College of Medicine

**Keywords:** Iceland, *Streptococcus pneumoniae*, adults, epidemiology, lower respiratory tract, molecular epidemiology, pneumococcus, pneumonia, vaccination

## Abstract

The introduction of pneumococcal conjugate vaccines (PCVs) into childhood vaccination programs has reduced carriage of vaccine serotypes and pneumococcal disease. The 10-valent PCV was introduced in Iceland in 2011. The aim of this study was to determine PCV impact on the prevalence of serotypes, genetic lineages, and antimicrobial-resistant pneumococci isolated from the lower respiratory tract (LRT) of adults. Pneumococci isolated between 2009 and 2017 at the Landspitali University Hospital were included (*n* = 797). The hospital serves almost three-quarters of the Icelandic population. Isolates were serotyped and tested for antimicrobial susceptibility, and the genome of every other isolate collected between 2009 and 2014 was sequenced (*n* = 275). Serotypes and multilocus sequence types (STs) were extracted from the genome data. Three study periods were defined, 2009 to 2011 (PreVac), 2012 to 2014 (PostVac-I), and 2015 to 2017 (PostVac-II). The total number of isolates and vaccine-type (VT) pneumococci decreased from PreVac to PostVac-II (*n* = 314 versus *n* = 230 [*p* = 0.002] and *n* = 170 versus *n* = 33 [*p* < 0.001], respectively), but non-vaccine-type (NVT) pneumococci increased among adults 18 to 64 years old (*n* = 56 versus *n* = 114 [*p* = 0.008]). Serotype 19F decreased in the PostVac-II period; these isolates were all multidrug resistant (MDR) and were members of the Taiwan^19F^-14 PMEN lineage. Serotype 6A decreased among adults ≥65 years old in the PostVac-II period (*p* = 0.037), while serotype 6C increased (*p* = 0.021) and most serotype 6C isolates were MDR. Nonencapsulated Streptococcus pneumoniae (NESp) isolates increased among adults 18 to 64 years old in the PostVac-II period, and the majority were MDR (*p* = 0.028). An overall reduction in the number of LRT samples and pneumococcus-positive cultures and significant changes in the serotype distribution became evident within 4 years, thereby demonstrating a significant herd effect.

## Abstract (precis)

The introduction of pneumococcal conjugate vaccines (PCVs) into childhood vaccination programs has reduced carriage of vaccine serotypes and pneumococcal disease. The 10-valent PCV was introduced in Iceland in 2011.

## INTRODUCTION

Pneumococcus is an important human pathogen that causes significant morbidity and mortality worldwide (1). It is one of the most important human pathogens in community-acquired and nosocomial pneumonia, causing an estimated two-thirds of all cases of bacterial pneumonia and resulting in hospitalization and death among older adults (2–6). One of its main virulence factors is a polysaccharide capsule (serotype), and nearly 100 different serotypes have been identified to date (7, 8).

Pneumococcal conjugate vaccines (PCVs) have been included in the infant vaccination program of more than 100 countries (9), which has resulted in a significant reduction in disease and circulating vaccine serotypes within those countries. Protection of the unvaccinated adult population through herd immunity is generally observed later than vaccine-induced immunity among the pediatric population (10–12). Importantly, serotype replacement and the circulation of antimicrobial-resistant pneumococcal lineages expressing non-vaccine serotypes can also occur (13–16).

In April 2011, the 10-valent PCV (PHiD-CV, Synflorix; GSK) was introduced into the national infant immunization program in a 2 + 1 vaccine schedule, without catch-up vaccination. The vaccine directly targets serotypes 1, 4, 5, 6B, 7F, 9V, 14, 18C, 19F, and 23F, but the potential cross-protection against vaccine-related serotypes 6A and 19A seems to vary among studies (17–21). Pneumococcal vaccines had not previously been a part of the routine infant immunization program in Iceland. In this study, we analyzed the impact of PHiD-CV implementation among pneumococci recovered from the lower respiratory tract (LRT) of adult patients. The distribution of pneumococcal serotypes and genetic lineages along with changes in antimicrobial resistance rates were assessed before and after vaccine implementation.

## MATERIALS AND METHODS

### Study population and bacterial isolates.

All pneumococci isolated from lower respiratory tract (LRT) samples taken from adults ≥18 years of age with suspected pneumonia and submitted to the Department of Clinical Microbiology at Landspitali University Hospital in Iceland between 2009 and 2017 were included in the study. When two or more pneumococcal isolates of the same phenotype (i.e., the same serotype and antimicrobial susceptibility pattern) were identified from the same patient within 30 days, they were considered to be from the same infection, and only the first isolate was included in the subsequent analyses.

The Department of Clinical Microbiology serves as the reference laboratory for the whole country and is the primary microbiology laboratory for the greater Reykjavík capital area. The study was divided into three different periods for the analyses, 3 years prior to vaccination (2009 to 2011, PreVac), 1 to 3 years postvaccination (2012 to 2014, PostVac-I), and 4 to 6 years postvaccination (2015 to 2017, PostVac-II).

The primary catchment area for the Landspitali University Hospital was considered to be within a 100-km driving distance from the hospital, and the population demographic information for this referral region was obtained from Statistics Iceland (www.statice.is). The population sizes of the referral region for adults ≥18 years of age (which includes over 70% of Icelandic adults ≥18 years old) were 170,042 (PreVac), 177,490 (PostVac-I), and 186,724 (PostVac-II). The prevalences of the pneumococcal isolates were calculated using the population size of the referral region as the denominator. The population sizes stratified by age group (18 to 64 and ≥65 years old) are shown in Table S1 in the supplemental material.

### Serotyping.

Serotypes were determined for all available isolates with ImmuLex pool antisera (State Serum Institute, Copenhagen, Denmark) and/or by a multiplex PCR (mPCR) method, which included 78 sets of serogroup/serotype-specific primer pairs. Serotypes of serogroup 6 were identified as previously described (22). Nonencapsulated Streptococcus pneumoniae (NESp) isolates (i.e., those that were negative for *cpsA* and positive for *lytA*) were tested for the *cpsB* gene, which is essential for encapsulation (23), using a previously published PCR method (24).

### DNA extraction, whole-genome sequencing, and phylogenetic analysis.

DNA extraction and whole-genome sequencing (WGS) were performed as previously described on every other pneumococcal isolate from the years 2009 to 2014 (22). Multilocus sequence types (STs) and clonal complexes (CCs) were defined in the standard manner. Genome annotation, gene clustering, and sequence alignments were performed using Prokka and Roary. FastTree and ClonalFrameML were used to reconstruct the phylogenetic tree as described in our previous paper (22). Note that a core genome threshold of 99.6% was calculated for this data set.

### Antibiotic susceptibility testing.

All isolates were tested for antimicrobial susceptibility to chloramphenicol, erythromycin, tetracycline, trimethoprim-sulfamethoxazole, and clindamycin using disk diffusion tests. Oxacillin disks (1 µg) were used to screen for penicillin resistance. E-tests were used to measure the MIC (bioMérieux, France) of penicillin and ceftriaxone on oxacillin-resistant isolates. Multidrug resistance (MDR) was defined as nonsusceptibility to three or more classes of antimicrobials (regardless of penicillin susceptibility). Susceptibility testing was performed according to the methods and criteria of the European Committee on Antimicrobial Susceptibility Testing (EUCAST) (25).

### Statistical analyses.

A likelihood ratio test (26) was used to test the null hypothesis of equality when comparing the rate (*r*_1_) of a certain serotype, CC, or ST in a given age group PreVac to the rate (*r*_2_) of the same serotype, CC, or ST in the same age group in PostVac-II (for serotypes) and PostVac-I (for CC/ST). For the CCs and STs, PreVac was compared to PostVac-I (2012 to 2014), as there was no genome sequencing done on pneumococcal isolates after 2014. The two-sided Fisher’s exact test was used to calculate the *p* values for antimicrobial resistance by using R version 3.3.2. The level of significance for all tests was ≤0.05. Simpson’s diversity index was calculated to assess the change in ST diversity after vaccine implementation (27).

### Ethics.

This study was approved by The National Bioethics Committee (VSNb2013010015/03.07) and the appropriate authorities at Landspitali University Hospital in Iceland.

## RESULTS

### Demographics.

**FIG 1:**
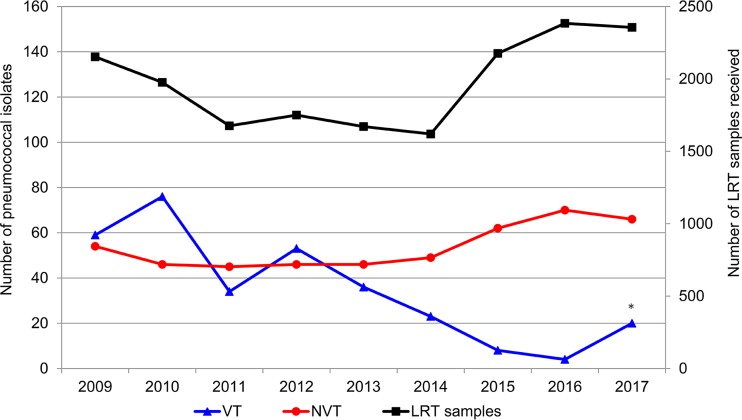
Annual number of isolates belonging to serotypes targeted by PHiD-CV (vaccine-type [VT]), serotypes not targeted by PHiD-CV (non-vaccine-type [NVT]), and lower respiratory tract (LRT) samples received by the laboratory. *, Seven isolates of serotype 19F were detected in the same patient in 2017.

The laboratory received 17,762 samples during the study period (Fig. 1). This yielded 814 pneumococcal isolates, of which 17 isolates were not stored or were nonviable, leaving 797 isolates for further analyses. About 10% of the LRT samples originated from adults residing outside of the primary catchment area. The total number of pneumococcal isolates decreased significantly from 314 (184.7/100,000 adults) PreVac to 230 (123.2/100,000 adults) PostVac-II (*p* = 0.002; Table 1). The median age of the patients with confirmed pneumonia was 75.2 years. More than half (430/797, 54.0%) of all isolates obtained were from patients ≥65 years of age, and in this age group, the total number of isolates decreased from 191 (719.8/100,000 adults aged ≥65 years) in the PreVac period to 102 (314.7/100,000 adults aged ≥65 years) in the PostVac-II period (*p* < 0.001; Table 2).

**TABLE 1:** Serotype distribution for each study year, 2009 to 2017, in adults ≥18 years of age

Serotype	No. of isolates per yr									PreVac isolates (2009–2011)		PostVac-I isolates (2012–2014)		PreVac vs PostVac-I *p* value	PostVac-II isolates (2015–2017)		PreVac vs PostVac-II *p* value
	2009	2010	2011	2012	2013	2014	2015	2016	2017	No.	No. per 100,000	No.	No. per 100,000		No.	No. per 100,000	
3	11	9	5	9	3	8	6	7	7	25	14.7	20	11.3	0.558	20	10.7	0.485
4	0	0	1	0	0	0	1	0	0	1	0.6	0	0	Nc^*a*^	1	0.5	0.965
6A	10	4	4	5	4	0	1	1	1	18	10.6	9	5.1	0.221	3	1.6	0.016
6B	9	10	2	11	7	3	3	3	1	21	12.3	21	11.8	0.927	7	3.7	0.052
6C	0	1	0	1	1	0	2	7	5	1	0.6	2	1.1	0.719	14	7.5	0.021
7F	0	2	0	1	0	0	2	0	0	2	1.2	1	0.6	0.683	2	1.1	0.951
8	0	1	0	0	0	0	0	0	0	1	0.6	0	0	Nc	0	0	Nc
9V	3	4	2	2	0	0	0	0	0	9	5.3	2	1.1	0.136	0	0	Nc
9A	1	0	1	0	1	1	0	0	0	2	1.2	2	1.1	0.977	0	0	Nc
9N	0	1	0	0	0	2	1	1	2	1	0.6	2	1.1	0.136	4	2.1	Nc
10	0	0	1	0	0	0	0	0	0	1	0.6	0	0	Nc	0	0	Nc
11A	5	6	3	2	6	3	7	4	9	14	8.2	11	6.2	0.641	20	10.7	0.617
14	8	7	0	3	3	1	0	0	1	15	8.8	7	3.9	0.229	1	0.5	0.008
15A	1	1	0	1	3	1	2	2	4	2	1.2	5	2.8	0.470	8	4.3	0.230
15B/C	3	0	5	3	5	3	4	6	1	8	4.7	11	6.2	0.694	11	5.9	0.749
16F	2	3	0	1	1	0	0	1	0	5	2.9	2	1.1	0.426	1	0.5	0.231
17F	0	0	1	0	0	1	0	0	0	1	0.6	1	0.6	0.984	0	0	Nc
18B	0	0	1	0	0	0	0	0	0	1	0.6	0	0	Nc	0	0	Nc
18C	2	0	0	0	0	0	0	0	0	2	1.2	0	0	Nc	0	0	Nc
19F	30	45	24	29	23	15	1	1	18^*b*^	99	58.2	67	37.7	0.068	20^*b*^	10.7	<0.001
19A	5	3	2	3	4	6	4	5	3	10	5.9	13	7.3	0.730	12	6.4	0.891
20	0	0	1	0	0	0	0	0	0	1	0.6	0	0	Nc	0	0	Nc
21	1	0	0	1	1	0	1	1	4	1	0.6	2	1.1	0.719	6	3.2	0.217
22F	4	3	5	5	1	3	5	4	1	12	7.1	9	5.1	0.619	10	5.4	0.670
23F	8	11	7	7	4	4	2	0	0	26	15.3	15	8.5	0.219	2	1.1	0.001
23A	3	0	0	1	1	2	5	2	6	3	1.8	4	2.3	0.832	13	7.0	0.111
23B	0	1	3	0	2	3	5	4	1	4	2.4	5	2.8	0.859	10	5.4	0.337
24F	0	0	0	0	0	0	1	1	0	0	0	0	0	Nc	2	1.1	Nc
31	0	1	0	0	0	0	0	0	0	1	0.6	0	0	Nc	0	0	Nc
33F	2	1	1	3	0	0	1	1	0	4	2.4	3	1.7	0.774	2	1.1	0.536
34	1	0	0	0	0	0	1	0	0	1	0.6	0	0	Nc	1	0.5	0.965
35F	0	1	1	3	3	4	1	3	3	2	1.2	10	5.6	0.123	7	3.7	0.298
35B	2	1	3	4	1	2	3	7	4	6	3.5	7	3.9	0.895	14	7.5	0.289
Not determined^*c*^	0	1	0	0	1	2	0	0	4	1	0.6	3	1.7	0.518	4	2.1	0.396
NESp^*d*^	2	5	6	4	7	8	11	13	11	13	7.6	19	10.7	0.534	35	18.7	0.054
Total	113	122	79	99	82	72	70	74	86	314	184.7	253	142.5	0.043	230	123.2	0.002
VT^*e*^	59	77	34	53	37	23	9	4	20	170	100.0	113	63.7	0.013	33	17.7	<0.001
NVT^*f*^	54	45	45	46	45	49	61	70	66	144	84.7	140	78.9	0.693	197	105.5	0.184
All LRT samples	2,153	1,976	1,676	1,750	1,671	1,620	2,176	2,384	2,356	5,805	3,413.9	5,041	2,840.2	<0.001	6,916	3,703.9	0.003
Percentage of LRT samples positive for pneumococci	5.2	6.2	4.7	5.7	4.9	4.4	3.2	3.1	3.7	5.4		5.0			3.3		

a Nc, not calculated.

b Seven isolates of serotype 19F were detected in the same patient in 2017.

c Serotypes other than those included in the multiplex PCR panel of the study.

d NESp, nonencapsulated S. pneumoniae.

e Serotypes detected in the study that are targeted by PHiD-CV (4, 6B, 7F, 9V, 14, 18C, 19F, and 23F).

f Serotypes detected in the study that are not targeted by PHiD-CV.

**TABLE 2:** Serotype distribution within each age group during the PreVac (2009 to 2011), PostVac-I (2012 to 2014), and PostVac-II (2015 to 2017) periods in LRT samples

Serotype	18–64 yrs								≥65 yrs							
	PreVac isolates (2009–2011)		PostVac-I isolates (2012–2014)		PreVac vs PostVac-I *p* value	PostVac-II isolates (2015–2017)		PreVac vs PostVac-II *p* value	PreVac isolates (2009–2011)		PostVac-I isolates (2012–2014)		PreVac vs PostVac-I *p* value	PostVac-II isolates (2015–2017)		PreVac vs PostVac-II *p* value
	No.	No. per 100,000	No.	No. per 100,000		No.	No. per 100,000		No.	No. per 100,000	No.	No. per 100,000		No.	No. per 100,000	
3	9	6.3	9	6.1	0.963	11	7.1	0.851	16	60.3	11	37.7	0.425	9	27.8	0.209
4	1	0.7	0	0	Nc^*a*^	1	0.6	0.973	0	0	0	0	Nc	0	0	Nc
6A	6	4.2	2	1.3	0.325	1	0.6	0.170	12	45.2	7	24.0	0.371	2	6.2	0.037
6B	3	2.1	13	8.8	0.095	5	3.2	0.688	18	67.8	8	27.4	0.143	2	6.2	0.005
6C	0	0	2	1.3	Nc	7	4.5	Nc	1	3.8	0	0	Nc	7	21.6	0.191
7F	1	0.7	1	0.7	0.988	1	0.6	0.973	1	3.8	0	0	Nc	1	3.1	0.926
8	0	0	0	0	Nc	0	0	Nc	1	3.8	0	0	Nc	0	0	Nc
9V	2	1.4	2	1.3	0.983	0	0	Nc	7	26.4	0	0	Nc	0	0	Nc
9A	0	0	0	0	Nc	0	0	Nc	2	7.5	2	6.9	0.950	0	0	Nc
9N	0	0	1	0.7	Nc	3	1.9	Nc	1	3.8	1	3.4	0.965	1	3.1	0.926
11A	7	4.9	6	4.0	0.824	11	7.1	0.601	7	26.4	5	17.1	0.665	9	27.8	0.946
14	7	4.9	3	2.0	0.379	0	0	Nc	8	30.1	4	13.7	0.381	1	3.1	0.068
15A	0	0	4	2.7	Nc	6	3.9	Nc	2	7.5	1	3.4	0.662	2	6.2	0.895
15B/C	4	2.8	3	2.0	0.781	9	5.8	0.400	4	15.1	8	27.4	0.508	2	6.2	0.482
16F	0	0	0	0	Nc	1	0.6	Nc	5	18.8	2	6.9	0.401	0	0	Nc
17F	1	0.7	1	0.7	0.988	0	0	Nc	0	0	0	0	Nc	0	0	Nc
18B	0	0	0	0	Nc	0	0	Nc	1	3.8	0	0	Nc	0	0	Nc
18C	1	0.7	0	0	Nc	0	0	Nc	1	3.8	0	0	Nc	0	0	Nc
19F	39	27.2	22	14.8	0.126	7	4.5	0.001	60	226.1	45	154.3	0.199	13^*b*^	40.1	<0.001
19A	5	3.5	6	4.0	0.973	7	4.5	0.765	5	18.8	7	24.0	0.784	5	15.4	0.835
20	0	0	0	0	Nc	0	0	Nc	1	3.8	0	0	Nc	0	0	Nc
21	0	0	2	1.3	Nc	4	2.6	Nc	1	3.8	0	0	Nc	2	6.2	0.786
22F	8	5.6	7	4.7	0.525	5	3.2	0.524	4	15.1	2	6.9	0.536	5	15.4	0.982
23F	14	9.8	6	4.0	0.264	0	0	Nc	12	45.2	9	30.9	0.566	2	6.2	0.037
23A	2	1.4	3	2.0	0.983	6	3.9	0.375	1	3.8	1	3.4	0.965	7	21.6	0.191
23B	1	0.7	3	2.0	0.715	4	2.6	0.388	3	11.3	2	6.9	0.715	6	18.5	0.638
24F	0	0	0	0	Nc	1	0.6	Nc	0	0	0	0	Nc	1	3.1	Nc
31	1	0.7	0	0	Nc	0	0	Nc	0	0	0	0	Nc	0	0	Nc
33F	0	0	3	2.0	Nc	2	1.3	Nc	4	15.1	0	0	Nc	0	0	Nc
34	0	0	0	0	Nc	1	0.6	Nc	1	3.8	0	0	Nc	0	0	Nc
35F	1	2.1	5	3.4	0.272	2	1.3	0.731	1	3.8	5	17.1	0.293	5	15.4	0.332
35B	3	2.1	2	1.3	0.749	7	4.5	0.441	3	11.3	5	17.1	0.703	7	21.6	0.522
Not determined^*c*^	1	2.1	3	2.0	0.514	1	0.6	0.973	1	3.8	0	0	NC	3	9.0	0.585
NESp^*d*^	6	4.2	7	4.7	0.886	25	16.2	0.028	7	26.4	12	41.1	0.532	10	30.9	0.833
Total	123	85.7	116	78.2	0.641	128	82.9	0.864	191	719.8	137	469.8	0.011	102	314.7	<0.001
VT^*e*^	67	46.7	47	31.7	0.176	14	9.1	<0.001	107	403.2	65	222.9	0.022	19	61.7	<0.001
NVT^*f*^	56	39.0	69	46.5	0.519	114	73.9	0.008	84	316.6	72	246.9	0.201	83	253.0	0.367

a Nc, not calculated.

b Seven isolates of serotype 19F were detected in the same patient in 2017.

c Serotypes other than those included in the multiplex PCR panel of the study.

d NESp, nonencapsulated S. pneumoniae.

e Serotypes detected in the study that are targeted by PHiD-CV (4, 6B, 7F, 9V, 14, 18C, 19F, and 23F).

f Serotypes detected in the study that are not targeted by PHiD-CV.

### Serotyping.

Among all 797 pneumococcal isolates, 789 (99.0%) were successfully serotyped, but 8 isolates were of serotypes other than those included in the mPCR scheme and were not characterized further. Overall, 31 serotypes were detected, 28 in the PreVac period and 24 each in the PostVac-I and PostVac-II periods. Overall, isolates of serotypes 4, 6B, 7F, 9V, 14, 18C, 19F, and 23F, which are targeted by PHiD-CV (vaccine type [VT]), decreased significantly between the PreVac and PostVac-II periods (*p* < 0.001; Table 1). VT serotypes 1 and 5 were not detected in the study. The prevalence of isolates with serotypes not targeted by PHiD-CV (non-vaccine type [NVT]) did not change significantly (*p* = 0.184; Table 1). VTs were most prevalent in 2010 (77/122; 63.1%) and least prevalent in 2016 (4/74; 5.4%) (Table 1 and Fig. 1).

Overall, among vaccine-related serotypes 6A and 19A, the frequency of serotype 19A did not change during the study period (PreVac, *n* = 10; PostVac-I, *n* = 13; and PostVac-II, *n* = 12), but serotype 6A decreased significantly from the PreVac to PostVac-II period (*n* = 18 versus *n* = 3, respectively; *p* = 0.016; Table 1). Notably, serotype 6C increased significantly from PreVac to PostVac-II (*n* = 1 versus *n* = 14, respectively; *p* = 0.021; Table 1).

Analyses by age group revealed that while the prevalence of VT pneumococci decreased significantly in both age groups, the prevalence of NVT and NESp isolates increased significantly in adults aged 18 to 64 years (*p* = 0.008 and 0.028, respectively). There was a nonsignificant decrease in the incidence of serotype 3 in the ≥65-year age group (from 60.3/100,000 adults in the PreVac period to 27.8/100,000 adults in the PostVac-II period). There was no significant change in the overall prevalence of NVT or NESp isolates in the ≥65-year age group (Table 2).

### CC/MLST.

Between 2009 and 2014, 567 pneumococcal isolates were recovered and 275 (48.5%) of these isolates were sequenced, among which 41 different CCs (31 CCs PreVac and 32 CCs PostVac-I) and 73 STs (56 STs PreVac and 47 STs PostVac-I) were detected. There was no difference in the ST diversity between the two periods (Simpson’s diversity indexes of the STs were 0.97 PreVac and 0.96 PostVac-I).

**FIG 2:**
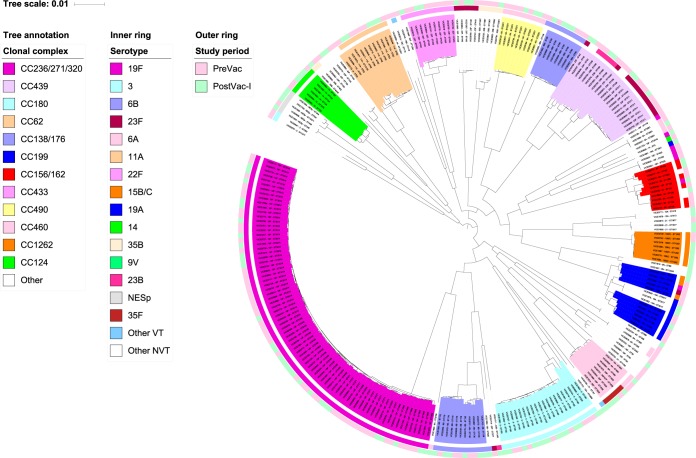
Midrooted phylogenetic tree, created from 1,221 full-length coding loci found in 99.6% of 275 genomes from LRT samples, annotated with CC designations. Serotypes (inner circle) are presented using the same colors as the appropriate CC where possible. Study periods (outer circle) are also presented.

A phylogenetic tree was created with concatenated sequences of 1,130 full-length coding loci found in 99.6% of the pneumococcal genomes. The tree was annotated with CC designations and serotypes (Fig. 2).

The most prevalent CC in both study periods was CC236/271/320^19F^, which comprised 31.2% (49/157 [28.8/100,000 adults]) of the PreVac pneumococci, but this CC decreased to 24.6% (29/118 [16.3/100,000 adults]) in the PostVac-I period (*p* = 0.010) (Fig. 2; see also Table S3 in the supplemental material). Minimal and insignificant changes in the prevalence of other CCs were observed, although the prevalence of CC433^22F^ decreased in the PostVac-I period (*p* = 0.042) (Fig. 2; Table S3).

### Antimicrobial resistance.

Approximately one-third of pneumococci were resistant to penicillin and/or erythromycin in all three study periods, and there was no significant change in those resistance rates over time (Table 3; see also Table S2 in the supplemental material). MDR pneumococci decreased from 34.4% in the PreVac period to 23.9% in the PostVac-II period (*p* = 0.010; Table 4). Prior to PHiD-CV implementation, 87.9% (102/116) of isolates that were penicillin nonsusceptible were also MDR, but this was reduced to 72.9% (51/70; *p* = 0.016) in the PostVac-II period (Table 5). This reduction was related mainly to the decrease in MDR serotype 19F pneumococci.

**TABLE 3:** PNSP serotypes during the PreVac (2009 to 2011) and PostVac-II (2015 to 2017) periods

PNSP serotype	PreVac isolates		PostVac-II isolates		*p* value
	No.	%	No.	%	
6A	0	0	1	1.4	0.458
6B	8	6.9	5	7.1	1.000
6C	0	0	5	7.1	0.007
9V	4	3.4	0	0	Nc^*a*^
9A	1	0.9	0	0	1.000
11A	0	0	1	1.4	0.376
14	3	2.6	1	1.4	1.000
15A	0	0	5	7.1	0.007
15B/C	0	0	1	1.4	0.376
19F	91	78.4	20^*b*^	28.6	<0.001
19A	2	1.7	0	0	Nc
22F	0	0	2	2.9	0.209
23F	1	0.9	0	0	1.000
23A	0	0	3	4.3	0.094
23B	0	0	3	4.3	0.094
35B	0	0	6	8.6	0.003
Not determined^*c*^	1	0.9	0	0	1.000
NESp^*d*^	5	4.3	17	24.3	<0.001
Total PNSP	116	100	70	100	0.121
Total pneumococcal isolates	314	36.9	230	30.4	0.002

a Nc, not calculated.

b Seven isolates of serotype 19F were detected in the same patient in 2017.

c Serotypes other than those included in the multiplex PCR panel of the study.

d NESp, nonencapsulated S. pneumoniae.

**TABLE 4:** MDR pneumococcal serotypes during the PreVac (2009 to 2011) and PostVac-II (2015 to 2017) periods

MDR serotype	PreVac isolates		PostVac-II isolates		*p* value
	No.	%	No.	%	
6A	1	0.9	0	0	1.000
6B	9	8.3	5	9.1	1.000
6C	1	0.9	9	16.4	<0.001
14	2	1.9	1	1.8	1.000
15A	1	0.9	5	9.1	0.017
15B/C	0	0	1	1.8	0.337
19F	87	80.6	20^*a*^	36.4	<0.001
23F	1	0.9	0	0	1.000
23A	0	0	1	1.8	0.337
35B	0	0	1	1.8	0.337
Not determined^*b*^	1	0.9	0	0	1.000
NESp^*c*^	5	4.6	12	21.8	<0.001
Total MDR serotypes	108	100	55	100	0.010
Total pneumococcal isolates	314	34.4	230	23.9	0.002

a Seven isolates of serotype 19F were detected in the same patient in 2017.

b Serotypes other than those included in the multiplex PCR panel of the study.

c NESp, nonencapsulated S. pneumoniae.

**TABLE 5:** PNSP serotypes that were also MDR PreVaC (2009 to 2011) and PostVac-II (2015 to 2017)

PNSP/MDR serotype	PreVac isolates		PostVac-II isolates		*p* value
	No.	%	No.	%	
6B	8	6.9	5	7.1	1.000
6C	0	0	5	7.1	0.007
14	2	1.7	1	1.4	1.000
15A	0	0	5	7.1	0.007
15B/C	0	0	1	1.4	0.376
19F	87	75.0	20^*a*^	28.6	<0.001
23A	0	0	1	1.4	0.376
35B	0	0	1	1.4	0.376
Not determined^*b*^	1	0.9	0	0	1.000
NESp^*c*^	4	3.4	12	17.1	0.002
Total PNSP/MDR	102	87.9	51	72.9	0.016
Total PNSP	116	100	70	100	0.121

a Seven isolates of serotype 19F were detected in the same patient in 2017.

b Serotypes other than those included in the multiplex PCR panel of the study.

c NESp, nonencapsulated S. pneumoniae.

Serotype 19F isolates were the most prevalent penicillin-nonsusceptible pneumococci (PNSP) and MDR pneumococci in all three study periods. A total of 78.4% of PNSP and 80.6% of MDR pneumococci recovered in the PreVac period were serotype 19F, but this decreased to 28.6% and 36.4%, respectively, in the PostVac-II period (*p* < 0.001 for both; Tables 3 and 4). Nearly all (91.8%) of the sequenced PNSP serotype 19F isolates were members of the internationally distributed MDR lineage CC236/271/320^19F^ (Taiwan^19F^-14; Table 3 and Fig. 2; see also Table S3 in the supplemental material).

There were three serotypes, 6C, 15A, and 35B, that were not associated with penicillin resistance in the PreVac period, but each serotype described 7 to 9% of the PNSP by the PostVac-II period (Table 3). Similarly, there were changes among the MDR pneumococci of all three of these serotypes; in particular, there were significant increases in MDR serotype 6C and 15A pneumococci (16.4% [*n* = 9] and 9.1% [*n* = 5] PostVac-II, respectively) (Tables 3 to 5). Among all PNSP isolates, NESp isolates increased from 4.3% to 24.3% from the PreVac period to the PostVac-II period (*p* < 0.001), of which 17.1% were also MDR (*p* = 0.002; Tables 3 to 5). The overall number of isolates of serotypes 6C, 15A, and 35B and that of the NESp isolates was relatively low, and only every other isolate from 2009 to 2014 was chosen for genome sequencing. Therefore, only a few of each of these pneumococci were selected for genome sequencing, and it is difficult to draw any major conclusions about the genetic lineages associated with these PNSP or MDR pneumococci, except to say that the characterized STs corresponded to widely distributed genetic lineages such as ST344^NT^ and ST315^6B^ (Fig. 2; Table S3).

## DISCUSSION

The results of this study demonstrated an indirect (herd) effect of PHiD-CV among adults by decreasing the overall number of LRT samples and the proportion of those samples that were positive for pneumococci and by causing a reduction in the proportion of pneumococci that were of vaccine serotypes. The vaccine-induced herd effect leading to a reduction in the incidence of pneumococcal serotypes in unvaccinated children and adults has been widely studied for invasive pneumococcal disease (IPD) (10, 28–31), but few studies have documented a herd effect on vaccine serotypes in adults with pneumonia (11, 32).

High vaccine coverage (>70 to 80%) leads to extensive herd protection in a population (33), which becomes evident in the adult population within a few years following vaccine implementation (10, 12). At the beginning of the PostVac-II period, over 97% of Icelandic children <5 years of age were fully vaccinated (34). We assessed the differences in the prevalences of pneumococcal serotypes between two postimplementation periods by comparing the PreVac period (2009 to 2011) to both the PostVac-I (2012 to 2014) and PostVac-II (2015 to 2017) periods. The relatively rapid establishment of herd protection and the decline of VTs in adults (29, 30, 33) could partly be explained by the high vaccine uptake in Iceland, a country where vaccines are generally well accepted (35). However, serotype replacement of NVTs has been observed where PCVs have been implemented (36–38), and this was also the case in our study, although serotype replacement was significant only in adults aged 18 to 64 years.

The penicillin-nonsusceptible and multidrug-resistant serotype 19F isolates were all members of the globally distributed CC236/271/320^19F^ lineage. Serotype 19F was the most prevalent PNSP/MDR serotype in all study periods, although it decreased significantly in the PostVac-II period. The prevalence of PNSP/MDR serotype 19F was unusually high in 2017 compared to that in the two previous years, but this was partly because one 77-year-old immunodeficient patient contributed 7 of the 18 isolates detected that year. However, fluctuations in the prevalence of serotype 19F are also known to occur following PCV introduction, and this will need to be monitored in Iceland going forward (11, 38). Adult infections are frequently a reflection of nasopharyngeal carriage among young children (31), but interestingly, serotype 19F was not detected in 2017 among healthy Icelandic children or in Icelandic children with acute otitis media (22). Therefore, it is possible that older children and/or older adults can serve as a reservoir for serotype 19F, and this maintains serotype 19F in the unvaccinated population after vaccine introduction. Serotypes 1 and 5 were not detected in this study. These serotypes are rare in Iceland. Serotype 1 was last detected in 2012, serotype 5 was last detected in 1996, and both serotypes were recovered from patients with IPD (S. J. Quirk, G. Haraldsson, M. Á. Hjálmarssdóttir, H. Erlendsdóttir, and K. G. Kristinsson, unpublished data).

Among vaccine-related serotypes, the prevalence of serotype 6A decreased significantly in the second PostVac period even though it is not a direct target of the vaccine, and other countries that have implemented PHiD-CV have also described similar results (18, 20). Furthermore, in our previous study, the prevalence of serotype 6A decreased in Icelandic children 1 to <4 years of age with acute otitis media (22). The possible cross-protection against serotype 19A through the serotype 19F conjugate has been widely debated (17–21); however, the Icelandic adult population did not appear to benefit from the childhood vaccinations against serotype 19A, since the incidence of serotype 19A did not change between the study periods. Before vaccine implementation in Iceland, serotype 19A was more commonly found in IPD and nasopharyngeal carriage than in non-IPD (acute otitis media and pneumonia), and serotype 19F was predominant in non-IPD (22, 39).

Serotypes 6C and 15A have been reported as upcoming PNSP and MDR serotypes following PCV introduction (13, 40, 41), but among adults in Iceland, these serotypes were detected only in low numbers in the PostVac periods of the study. Our group has also detected MDR isolates of serotype 6C that were members of CC315^6B/6C^ and ST386^6C^ (a double locus variant of PMEN Poland^6B^-20) among children (22, 39), but it remains to be seen whether CC315^6C^ will replace CC236/271/320^19F^ as a major MDR lineage in Iceland.

Notably, the prevalence of NESp isolates increased significantly, and they were the most frequently detected pneumococci in the PostVac-II period in adults aged 18 to 64 years. The opposite was seen in the United States, where NESp isolates decreased in adults between the ages of 50 and 64 years, with a parallel increase among adults ≥65 years of age (32).

Increased vaccine pressure caused by PCVs might open an environmental niche that NESp is adept to employ. This could explain the increased prevalence of NESp isolates in the PostVac-II period, or the increase might simply be a natural trend that reflects longer-term variation (42, 43). Our data support the need for continued surveillance of pneumococci in Iceland. It should be noted that some serotypes were found only in low numbers; therefore, the statistical power for detecting a difference in the frequency between the PreVac and PostVac periods among these serotypes was low.

Following the financial crisis in Iceland in 2008, physicians were advised to reduce test samples at the Landspitali University Hospital, and as a result, fewer LRT samples were received from 2010 to 2014. In the following years, when the effect of the crisis subsided, the number of LRT samples gradually increased again, but at the same time, the number of pneumococcal isolates continued to decrease. This decrease was particularly evident among patients aged ≥65 years, and PCV introduction into childhood immunization programs has been shown to be very beneficial for older adults (11, 33).

Most of the LRT samples in this study were sputum samples; thus, some pneumococcal isolates may represent colonization, although colonization among adults is rare (44), and sputum samples should have been collected only from patients with suspected pneumonia and not healthy adults. Therefore, using the number of positive pneumococcal isolates as a proxy for pneumococcal pneumonia could be considered a weakness. In general, the financial crisis mentioned above was the only known factor influencing sampling. Vaccine uptake area, guidelines, the health care system, and microbiological methods remained the same during the study period.

The herd effect became evident in our study 3 to 4 years after PHiD-CV implementation and was associated with significant changes in both the serotype distribution and the number of pneumococcal isolates cultured from the lower respiratory tract samples of adults. Pneumococcus was the most frequent pathogen recovered from adults with pneumonia in Iceland prior to vaccination (45); hence, the protection of older adults through pneumococcal vaccination and herd immunity is of the utmost importance.

## Supplementary Material

Supplemental file 1

Supplemental file 2

Supplemental file 3
