# Supplementary material for: Vaccination of Icelandic Children with the 10-Valent Pneumococcal Vaccine Leads to a Significant Herd Effect among Adults in Iceland
Source: J Clin Microbiol. 2019 Mar 28;57(4):e01766-18. doi: 10.1128/JCM.01766-18 (PMC6440763; doi:10.1128/JCM.01766-18)
Supplement: Supplemental file 1 [file JCM.01766-18-s0001.pdf]

Table S1. Number of adults within the uptake area, in all of Iceland and coverage according to each age group PreVac (2009-2011), PostVac-I (2012-2014) and PostVac-II (2015-2017).

Study period	Age group	Adults in uptake area	Adults in Iceland	Proportion of the Icelandic population
PreVac	18-64 years	143,507	199,718	72%
	≥65 years	26,535	38,138	70%
	≥18 years	170,042	237,856	72%
PostVac-I	18-64 years	148,326	199,544	74%
	≥65 years	29,164	39,188	74%
	≥18 years	177,490	238,732	74%
PostVac-II	18-64 years	154,313	199,948	77%
	≥65 years	32,411	40,391	80%
	≥18 years	186,724	240,339	78%
