# Supplementary material for: Vaccination of Icelandic Children with the 10-Valent Pneumococcal Vaccine Leads to a Significant Herd Effect among Adults in Iceland
Source: J Clin Microbiol. 2019 Mar 28;57(4):e01766-18. doi: 10.1128/JCM.01766-18 (PMC6440763; doi:10.1128/JCM.01766-18)
Supplement: Supplemental file 2 [file JCM.01766-18-s0002.pdf]

Table S2. Non-susceptibility to standard antimicrobials in the PreVac (2009-2011) and PostVac-II (2015-2017) periods in lower respiratory tract (LRT) samples.

**LRT**	**PreVac**		**PostVac-II**		
**Non-susceptibility to**	**n**	**%**	**n**	**%**	**p-value**
Penicillin	116	36.9	70	30.4	0.121
Erythromycin	118	37.6	77	33.5	0.366
Chloramphenicol	5	1.6	5	2.2	0.750
Tetracycline	109	34.7	62	27.0	0.062
Clindamycin	102	32.5	56	24.3	**0.045**
Trimethoprim-sulfamethoxazole	134	42.7	49	21.3	**<0.001**
**Total LRT isolates**	**314**		**230**		
