# Supplementary material for: Vaccination of Icelandic Children with the 10-Valent Pneumococcal Vaccine Leads to a Significant Herd Effect among Adults in Iceland
Source: J Clin Microbiol. 2019 Mar 28;57(4):e01766-18. doi: 10.1128/JCM.01766-18 (PMC6440763; doi:10.1128/JCM.01766-18)
Supplement: Supplemental file 3 [file JCM.01766-18-s0003.pdf]

Table S3. Serotypes, CCs and STs detected PreVac (2009-2011) and PostVac-I (2012-2014) in LRT samples. Arranged according to the most prevalent serotype, CC and ST detected PreVac.

LRT samples PreVac (2009-2011)			LRT samples PostVac-I (2012-2014)		
**Serotype (n)**	**CC (n)**	**ST (n: PMEN[a])**	**Serotype (n)**	**CC (n)**	**ST (n: PMEN)**
19F (54)	236/271/320 (49)	3014 (24: DLV[b] Taiwan^{19F}-14), 271 (8: DLV Taiwan^{19F}-14)	19F (31)	236/271/320[c] (29)	3014[d] (12: DLV Taiwan^{19F}-14), 9165 (8: DLV Taiwan^{19F}-14)
		9165 (7: DLV Taiwan^{19F}-14), 1968 (6: DLV Taiwan^{19F}-14)			271 (3: SLV Taiwan^{19F}-14), 1968 (3: DLV Taiwan^{19F}-14)
		10369 (2: DLV Taiwan^{19F}-14), 10376 (1), 13101 (1)			9458 (1: DLV Taiwan^{19F}-14)
	395 (3)	425 (3)			9828 (1), 10390 (1: DLV Taiwan^{19F}-14)
	199 (1)	199 (1: Netherlands^{15B}-37)		15 (1)	3016 (1)
	177 (1)	179 (1: SLV[e] Portugal^{19F}-21)		156/162 (1)	162 (1: SLV Spain^{9V}-3)
23F (12)	439 (8)	311 (6: DLV Tennessee^{23F}-4), 10348 (1: DLV Tennessee^{23F}-4), 10347 (1)	23F (7)	439 (4)	311 (3: DLV Tennessee^{23F}-4)
	392 (2), 199 (1)	440 (2), 199 (1: Netherlands^{15B}-37)			42 (1: DLV Tennessee^{23F}-4)
	361 (1)	277 (1)		392 (3)	440 (3)
6A (12)	490 (6), 460 (2)	2221 (6), 460 (2)	6A (6)	490 (3), 15 (1), 460 (1)	2221 (3), 3981 (1), 460 (1)
	15 (2), 395 (2)	3981 (2), 395 (2: SLV Portugal^{6A}-41)		9789 (1)	2756 (1)
3 (10)	180 (8)	180 (6: Netherlands^{3}-31), 13100 (1: SLV Netherlands^{3}-31)	3 (13)	180 (13)	180 (13: Netherlands^{3}-31)
		505 (1: DLV Netherlands^{3}-31), 13100 (1)			
	260 (1), 378 (1)	260 (1), 1377 (1)			
6B (10)	138/176 (5)	176 (3: DLV Poland^{23F}-16), 138 (2)	6B (11)	138/176 (6)	38 (3), 176 (3: DLV Poland^{23F}-16)
	90 (4), 146 (1)	90 (4: Spain^{6B}-2), 146 (1)		90 (4), 171 (1)	90 (4: Spain^{6B}-2), 1639 (1)
22F (8)	433 (8)	433 (8)	22F (3)	433[f] (2), 1294/4522 (1)	433[f] (2), 1294 (1)
11A (6)	62 (6)	62 (6: DLV Netherlands^{8}-33)	11A (5)	62 (5)	62 (4: DLV Netherlands^{8}-33), 13130 (1)
19A (6)	199 (5)	199 (3: Netherlands^{15B}-37), 667 (1: SLV Netherlands^{15B}-37)	19A (4)	3017 (2)	3017 (2)
		10360 (1: DLV Netherlands^{15B}-37)		199 (1)	199 (1: Netherlands^{15B}-37)
	3017 (1)	3017 (1)		Sing[g]1801 (1)	1801 (1)
14 (4)	124 (3)	124 (2: Netherlands^{14}-35), 1975 (1: SLV Netherlands^{14}-35)	14 (3)	124 (3)	124 (3: Netherlands^{14}-35)
	15 (1)	9 (1: England^{14}-9)			
35B (5)	1816 (4), 452 (1)	1967 (3), 10361 (1), 452 (1)	35B (2)	198 (1), 1816 (1)	4346 (1), 10361 (1)
9V (4)	156/162 (4)	156 (2: Spain^{9V}-3), 162 (1: SLV Spain^{9V}-3), 1269 (1: DLV Spain^{9V}-3)	9V (2)	156/162 (2)	162 (2: SLV Spain^{9V}-3)
15B/C (4)	199 (2), 1262 (2)	199 (2: Netherlands^{15B}-37), 1262 (2)	15B/C (6)	1262 (5), 199 (1)	1262 (5), 199 (1: Netherlands^{15B}-37)
23B (3)	439 (3)	439 (3: SLV Tennessee^{23F}-4)	23B (3)	439 (2), 338 (1)	439 (2: SLV Tennessee^{23F}-4), 1349 (1: DLV Colombia^{23F}-26)
23A (3)	439 (3)	436 (2: DLV Tennessee^{23F}-4), 42 (1: DLV Tennessee^{23F}-4)	23A (1)	439 (1)	42 (1: DLV Tennessee^{23F}-4)
16F (3)	30 (3)	30 (2), 2042 (1)	16F (1)	30 (1)	30 (1)
33F (2)	100 (2)	100 (2)	33F (2)	100 (2)	100 (2)
9A (2)	156/162 (2)	156 (1: Spain^{9V}-3), 1269 (1: DLV Spain^{9V}-3)	9A (1)	156/162 (1)	156 (1: Spain^{9V} -3)
8 (1)	62 (1)	53 (1: Netherlands^{8}-33)	8 (0)		
21 (1)	193 (1)	1877 (1: DLV Greece^{21}-30)	21 (2)	193 (2)	1877 (2: DLV Greece^{21}-30)
31 (1)	113 (1)	1766 (1)	31 (0)		
34 (1)	4878 (1)	10357 (1)	34 (0)		
18B (1)	113 (1)	110 (1: SLV Netherlands^{18C}-36)	18B (0)		
18C (1)	113 (1)	116 (1: SLV Netherlands^{18C}-36)	18C (0)		
35F (1)	460 (1)	1635 (1)	35F (4)	460 (4)	1635 (4)
6C (1)	315 (1)	386 (1: DLV Poland^{6B}-20)	6C (1)	315 (1)	386 (1: DLV Poland^{6B}-20)

15A (0)			15A (3)	193 (2), 3058 (1)	410 (2: SLV Greece^{21}-30), 4965 (1)
9N (0)			9N (2)	66 (2)	66 (1: SLV Tennessee^{14}-18), 10344 (1: DLV Tennessee^{14}-18)
7F (0)			7F (1)	191 (1)	191 (1: Netherlands^{7F}-39)
NESp[h] (1)	448 (1)	448 (1: USA^{NT}-43)	NESp (4)	344 (3)	344 (2: Norway^{NT}-42), 10371 (1:SLV Norway^{NT}-42)
				448 (1)	448 (1: USA^{NT}-43)

[a]PMEN: Pneumococcal molecular epidemiology network clone. [b]DLV: Double locus variant. [c]P-value=0.001, [d]P-value=0.030, [e]SLV: Single locus variant. [f]P-value=0.040. [g]Sing: Singleton.
[h]NESp: Non-encapsulated *S. pneumoniae.*
